# Training Community Health Workers to Manage Uncomplicated and Severe Malaria: Experience From 3 Rural Malaria-Endemic Areas in Sub-Saharan Africa

**DOI:** 10.1093/cid/ciw624

**Published:** 2016-12-06

**Authors:** Mohamadou Siribié, IkeOluwapo O. Ajayi, Jesca Nsungwa-Sabiiti, Chinenye Afonne, Andrew Balyeku, Catherine O. Falade, Zakaria Gansane, Ayodele S. Jegede, Lillian Ojanduru, Frederick O. Oshiname, Vanessa Kabarungi, Josephine Kyaligonza, Armande K. Sanou, Luc Sermé, Joëlle Castellani, Jan Singlovic, Melba Gomes

**Affiliations:** 1 Groupe de Recherche Action en Santé, Ouagadougou, Burkina Faso; 2 Child Health Division, Ministry of Health, Kampala, Uganda; 3 Department of Epidemiology and Medical Statistics; 4 Epidemiology and Biostatistics Research Unit, Institute of Advanced Medical Research and Training; 5 Department of Pharmacology and Therapeutics, College of Medicine; 6 Department of Sociology, Faculty of Social Sciences; 7 Department of Health Promotion and Education, Faculty of Public Health, College of Medicine, University of Ibadan, Nigeria; 8 Department of Health Services Research, School for Public Health and Primary Care, Maastricht University, The Netherlands; 9 UNICEF/UNDP/World Bank/WHO/Special Programme for Research & Training in Tropical Diseases, World Health Organization, Geneva, Switzerland

**Keywords:** malaria, ACTs, rectal artesunate, community health worker, training

## Abstract

***Background.*** Use of community health workers (CHWs) to increase access to diagnosis and treatment of malaria is recommended by the World Health Organization. The present article reports on training and performance of CHWs in applying these recommendations.

***Methods.*** Two hundred seventy-nine CHWs were trained for 3–5 days in Burkina Faso, Nigeria, and Uganda, and 19 were certified to diagnose and treat only uncomplicated malaria and 235 to diagnose and treat both uncomplicated and severe malaria. Almost 1 year after training, 220 CHWs were assessed using standard checklists using facility staff responses as the reference standard.

***Results.*** Training models were slightly different in the 3 countries, but the same topics were covered. The main challenges noticed were the low level of education in rural areas and the involvement of health staff in the supervision process. Overall performance was 98% (with 99% in taking history, 95% in measuring temperature, 85% for measuring respiratory rates, 98% for diagnosis, 98% for classification, and 99% for prescribing treatment). Young, single, new CHWs performed better than their older, married, more experienced counterparts.

***Conclusions.*** Training CHWs for community-based diagnosis and treatment of uncomplicated and severe malaria is possible with basic and refresher training and close supervision of CHWs’ performance.

***Clinical Trials Registration.*** ISRCTRS13858170.

Community health workers (CHWs) constitute an important resource in improving maternal and child health, when trained to bridge the gap in access to care in areas where formal health services are distant or limited [1, 2]. When well trained, supervised, and supported, CHWs have demonstrated reductions in severe malaria and mortality in their communities [3, 4]. Such has been their success that global malaria control recommendations now encourage their use to reduce malaria morbidity and mortality [5, 6].

Widespread CHW-based deployment of artemisinin-based combination therapies (ACTs) and rapid diagnostic tests (RDTs) is feasible and acceptable [7, 8, 9]. The World Health Organization (WHO) recommends that all treatment be based on parasitology [6] and encourages use of quality-assured malaria RDTs where microscopy is not practical to reduce unnecessary ACT treatment and identify patients who need alternative management [6, 10, 11].

Rectal artesunate is recommended as prereferral treatment for children who cannot take oral medication [6], and CHWs are increasingly being identified and trained to assess patients with signs of uncomplicated *and* severe illness in their communities as part of the Integrated Management of Childhood Illness (IMCI) approach [12, 13]. Although there is evidence from randomized controlled trials that CHWs can be successfully trained to assess and treat children with severe illness [14, 15], there is little evidence on how well they can be trained and how they perform on different components of training when routine scaling up is envisaged [12]. Children who require prereferral treatment with rectal artesunate are at high risk of death, with a range of symptoms from prostration to coma; urgent information is needed on the extent to which CHWs comply with training guidelines in routine deployment, both to reassure programs about what can be achieved in scaling up, as well as to improve training components that do not work well, or at all. The primary benefit of rectal artesunate is for those who need time to reach a referral facility. The risk of mortality is considerable if patients do not proceed to hospital; there is a threat of drug resistance if patients with malaria improve but are not given ACTs in the convalescent phase [16] and an added hazard that other serious infections are the true cause of illness in a prereferral patient [17, 18]. For many reasons therefore, referral advice to proceed immediately for further care requires emphasis.

In the context of a deployment study to scale up access to RDTs, ACTs, and rectal artesunate in 3 malaria-endemic countries of Africa [7], we assessed how well CHWs could be trained in IMCI [12, 13]. We report on our experience in training CHWs in Burkina Faso, Nigeria, and Uganda and on the performance of CHWs in assessing, classifying, and treating children according to the IMCI strategy.

## METHODS

### Study Settings

The study was conducted in rural malaria-endemic areas of Burkina Faso, Nigeria, and Uganda, as a component of a larger study to evaluate access to diagnostics and antimalarials provided by CHWs in their communities [7].

### Training Models

After providing information and receiving permission from local health authorities and communities to implement the study, community members were selected by their communities to serve as CHWs. A cascade system was adopted for training in Burkina Faso and Uganda: local facility–based health staff and CHW supervisors were trained as trainers by the investigative team and district health representatives. The pool of the CHW trainers was formed by local health staff, laboratory technicians under supervision by the principal investigators, and additional experts in clinical medicine and community health in Uganda. Thereafter, CHWs (and the inventory controller at the health facility in Burkina Faso) were trained by trainers. In Nigeria, the CHWs were selected by their communities but were trained by the investigative team. The training was conducted in the local languages Yoruba in Nigeria; English, Luganda, and Lunyankole in Uganda; and French and Dioula in Burkina Faso (see Supplementary Tables 1–5 for more details).

### Training Approach: Theoretical Versus Practical

In Burkina Faso, 60 CHWs were trained. Each training session had a maximum of 15 CHWs and lasted 3 days. CHWs were trained on the operational definition of malaria, the recognition of symptoms and signs of uncomplicated and severe malaria, use of thermometers, measurement of respiratory rates, RDT use, cassette storage, and waste disposal. Job aids were used. Treatment of uncomplicated malaria using ACTs and counseling of caregivers posttreatment, assessment of danger signs, different insertion methods for rectal artesunate, and referral advice for immediate transit, follow-up visits, the informed consent process, and documentation were covered. Practical sessions accompanied theory and included demonstration by facilitators, RDT practice, role plays, and discussion sessions. Supplementary Tables 1–5 provide further detail on the topics covered and the training tools used for each country.

In Nigeria, 55 female CHWs were trained, with a maximum of 28 CHWs trained per session. Training lasted 3 days. Topics were similar to that in Burkina Faso with sessions on malaria, its causes and transmission, signs and symptoms of uncomplicated and severe malaria, malaria diagnosis, demonstration of RDT using job aids, and preparation of thick smears. Role play and practical sessions on communication with caregivers/child, diagnosis and treatment of febrile children, and record-keeping were used. For the role plays, facilitators acted different scenarios (including severe and uncomplicated malaria situations) for each group of 3. There were interactive sessions on what to do, how to conduct tests, ACT dose, and referral acted in role plays so that observations could be shared and corrected.

In Uganda, training occurred over the course of 5 days with 3 days of theory and 2 days of practice (with respiratory rate counting, communicating with caregivers, practice of RDTs, counting respiratory rates with timers, communicating with caregivers about problems), and 164 CHWs were trained.

Training on suppository administration involved using a mannequin in Nigeria and pictorial materials on the different methods of insertion of the suppository, in the WHO manual and video of WHO on the prereferral treatment with rectal artesunate in Burkina Faso and Uganda. CHWs were also taught to use gloves when administering a suppository and the number of suppositories to insert according to the child's age. Quality control was implemented during the supervision of the CHW at home [19].

### Evaluation of the Training

In all countries, training was evaluated through pre–post tests using a standardized questionnaire and by observation of CHW practice on real patients by facilitators of training sessions. In Burkina Faso, 50 were certified to be involved in the implementation of the uncomplicated component; 34 passed the uncomplicated and severe components. Ten candidates were disqualified because of inability to read and to complete the different forms. In Nigeria, all trainers monitored CHW performance for all training components. Evaluation was subjective, but all trainers agreed that 4 out of 55 trained could not be certified. In Uganda, all 164 CHWs who were trained over the 5 days passed their certification test.

### Refresher Training

Different approaches for the refresher training were developed by each country team, focused on study procedures, documentation, the use and interpretation of RDT results, preparation of blood smears (Nigeria), and difficulties encountered (eg, calculation of the age and time). Regular monthly supervision of CHWs was implemented in Nigeria and Burkina Faso, aimed at assessing the conduct of CHWs’ activities according to protocol and guidelines in their home environment, to identify the challenges encountered and to address corrective actions. One refresher training was performed 6 months after the initial training in Burkina Faso, and quarterly refresher training was conducted in Nigeria and Uganda. Review of forms completed and drug accountability occurred at each supervisory visit with supply replenishment.

### Study Design and Participants

A narrative method was used to report on the training processes of CHWs and, for assessment of performance, cross-sectional surveys were carried out at the health facility level toward the end of the study (at 1 year of implementation) involving 220 CHWs including 47 in Burkina Faso, 35 in Nigeria, and 138 in Uganda. Variables assessed are summarized in Supplementary Table 4.

### Data Collection

Standard checklists were developed and pretested before use. Using this checklist, 1 physician and 1 nurse observed each CHW's practice with sick children at the health center/hospital. Parents/guardians of children aged <5 years were invited to participate in the study on arrival at the facility after registration and obtaining consent. Enrolled children were managed at the end of their participation by the physician. Any under-5 child attending the health center with a temperature or history of fever, with or without danger signs, or attending the health center for immunization and nutritional status checkup was eligible for participation. Each CHW was evaluated with 2 children in Burkina Faso and 1 child in Nigeria and Uganda.

### Data Management and Analysis

Data were double-entered into EpiData and analysis was done using *Stata Statistical Software: Release 11 & 14*. Indicators were measured for each part and given a uniform score of 1 for correct and 0 for incorrect. Scores were weighted by importance. The total score for each CHW ranged from 100% to 0%, computed as a percentage of the total maximum score expected. A cutoff of 90% was set as good performance; results from 60% to 89% were classified as medium; and results <60% were classified as poor. CHWs’ performance was summarized using descriptive statistics. Cohen κ was used to estimate CHW proﬁciency in reading RDT results, as well as counting respiratory rates and taking axillary temperature. Bivariate analysis was used to assess the association between the good performance CHWs’ sociodemographic characteristics.

### Ethics Statement

The study protocol was approved by the ethics review committee of WHO and approved by the local ethics committees of the different countries involved in the study. Participation was voluntary and participants were free to withdraw at any time.

## RESULTS

### Sociodemographic Characteristics of CHWs

A total of 47, 35, and 138 CHWs were evaluated in Burkina Faso, Nigeria, and Uganda, respectively. Demographic characteristics are outlined in Table 1. Men were predominant in Burkina Faso and women in Uganda and Nigeria; mean age was 38 years. All CHWs were literate. Farming was the main activity in Burkina Faso and Uganda whereas the CHWs in Nigeria were mainly traders. CHW service was >3 years in 57%, 37%, and 59% cases in Burkina Faso, Nigeria, and Uganda, respectively (Table 1).

**Table 1.** Sociodemographic Characteristics of Community Health Workers

Characteristic	Study Site			
	Burkina Faso	Nigeria	Uganda	Total
CHWs	47 (21.36)	35 (15.91)	138 (62.73)	220 (100)
Sex				
Male	37 (78.72)	0 (0)	36 (26.09)	73 (33.18)
Female	10 (21.28)	35 (100)	102 (73.91)	147 (66.82)
Mean age, y (SD)	36.48 (10.91)	40.57 (8.42)	39.06 (9.98)	38.33 (9.73)
Age group				
<30 y	17 (36.17)	1 (2.86)	19 (13.77)	37 (16.81)
30–45 y	20 (42.55)	27 (77.14)	84 (60.87)	131 (59.55)
>45 y	10 (21.28)	7 (20)	28 (20.29)	45 (20.45)
Unknown	0 (0)	0 (0)	7 (5.07)	7 (3.18)
Marital status				
Married	40 (85.10)	28 (80)	117 (84.78)	185 (84.09)
Not married	6 (12.77)	0 (0)	4 (2.9)	10 (4.55)
Divorced	0 (0)	3 (8.57)	0 (0)	3 (1.36)
Widower	1 (2.13)	4 (11.43)	0 (0)	5 (2.27)
Unknown	0 (0)	0 (0)	17 (12.32)	17 (7.72)
Level of education				
Local language literacy	2 (4.25)	0 (0)	0 (0)	2 (0.90)
Primary	24 (51.06)	17 (48.57)	44 (31.88)	85 (38.64)
Secondary	21 (44.68)	16 (45.71)	70 (50.72)	107 (48.64)
Tertiary	0 (0)	2 (5.71)	2 (1.45)	4 (1.82)
Unknown	0 (0)	0 (0)	22 (15.94)	22 (10)
Experience as CHW				
No experience	0 (0)	4 (11.43)	0 (0)	4 (1.82)
1–3 y	20 (42.55)	18 (51.43)	54 (39.13)	92 (41.82)
>3 y	27 (57.45)	13 (37.14)	81 (58.7)	121 (55)
Unknown	0 (0)	0 (0)	3 (2.17)	3 (1.36)
Main occupation				
Farmer/animal husbandry	42 (89.36)	5 (14.29)	105 (76.09)	152 (69.09)
Trader	4 (8.51)	24 (68.57)	15 (10.87)	43 (19.55)
Housewife	1 (2.13)	0 (0)	2 (1.4)	3 (1.36)
Other	0 (0)	6 (17.14)	12 (8.70)	18 (8.18)
Unknown	0 (0)	0 (0)	4 (2.9)	4 (1.82)

Data are presented as No. (%) unless otherwise indicated.

Abbreviations: CHW, community health worker; SD, standard deviation; y, year.

### Assessing, Classifying, Treating, and Counseling Children With Malaria (Score)

The performance of CHWs in the study sites was 99% for taking history, 95% for temperature, 85% measuring respiratory rates, and 98% for RDTs (Table 2). Temperature was taken in Burkina Faso and Nigeria. The performance score in Uganda for respiratory rates was <90%, as was the measurement of temperature in Nigeria. The scores of performance of CHWs in classifying and prescribing treatment were >90% in all study sites. Overall performance of CHWs by country was 99.8% in Burkina Faso, 94% in Nigeria, and 98% in Uganda.

**Table 2.** Performance of Community Health Workers’ Observed Child Assessments

Assessment	Item Weight	Burkina Faso	Nigeria	Uganda	Overall
Taking history		100 (658/658)	93.5 (229/245)	99.7 (963/966)	99.0 (1850/1869)
Asks the notion of hot body	4	100 (376/376)	88.6 (124/140)	100 (552/552)	98.5 (1052/1068)
Asks the child's age/DOB	3	100 (282/282)	100 (105/105)	99.3 (411/414)	99.6 (798/801)
Taking temperature	2	100 (188/188)	80 (56/70)	…	94.6 (244/258)
Using timer for respiratory rate	1	97.9 (92/94)	…	76.8 (106/138)	85.3 (198/232)
Proficiency in the use of RDT		99. 7 (1218/1222)	94.1 (428/455)	97.4 (1748/1794)	97.8 (3394/3471)
Waits before reading results	4	100 (376/376)	97.1 (136/140)	95.7 (528/552)	97.4 (1040/1068)
Interpretation of the result	3	100 (282/282)	97.1 (102/105)	97.1 (402/414)	98.1 (786/801)
Pricks the right finger	2	100 (188/188)	88.6 (62/70)	99.3 (274/276)	98.1 (524/534)
Blood and buffer placed in wells	4	98.9 (372/376)	91.4 (128/140)	98.6 (544/552)	97.8 (1044/1068)
Performance in classifying	4	100 (376/376)	91.2 (124/136)	98.5 (536/544)	98.1 (1036/1056)
Performance in prescribing treatment	4	100 (376/376)	100 (140/140)	96.6 (340/352)	98.6 (856/868)
Overall performance in above categories		99.8 (4784/4794)	93.6 (1634/1746)	97.7 (6404/6554)	97.9 (12822/13094)

Data are presented as no./No. (%).

Abbreviations: DOB, date of birth; RDT, rapid diagnostic test of malaria.

### Assessing Age, Fever, and Respiratory Rates

During history-taking of the ill child, all CHWs asked about the age of the children they saw, but their age calculations were recorded only in Burkina Faso. The mean age of the study children was 24.1 (SD, 13.85) months and 23.8 (SD, 13.53) months by CHWs and evaluators, respectively. Mean temperature recorded by CHWs was 38.3°C (SD, 1.80°C) and that reported by the observers was 38.4°C (SD, 1.71°C) (*t* = −0.1175; *P* = .9066). Temperature (≥37.5°C) recorded by the CHWs was 81.9% (77/94), which agreed well with observer recordings (κ = 1 and *P* < .0001). The readings of respiratory rates by CHWs were correct in 85.3% (198/232) of cases compared with observers as the gold standard. Rapid breathing, recorded only in Burkina Faso, was 24% (21/87) and 40% (35/87) according to the CHW and observers, respectively. However, there was an agreement between CHW and observers (κ = 0.642; *P* < .0001).

### Assessment of Danger Signs

Only 4.6% (8/175) of the study children had danger signs, and CHWs assessed these children reasonably well in comparison with danger signs noted by observers.

### Performance Using RDTs for Malaria Diagnosis

Although the proficiency of CHWs in the use of RDT was not 100% in the 3 study sites, the readings were almost in agreement with those of the observers (265/267) (κ = 0.941; *P* < .0001). Malaria positivity was 67% (180/267).

### Prescribing Treatment and Counseling of Caregivers

Based on the RDT results and observers’ judgement (the age of the child), CHWs correctly prescribed ACTs to 98% (167/171) of uncomplicated malaria cases; rectal artesunate was also correctly proposed to 100% of eligible patients (*non per os* malaria cases).

### Association Between CHWs’ Performance and Sociodemographic Characteristics

Overall, 53% of CHWs had a good performance in assessing, 96% in classifying, and 40% in treating and counseling children; overall good performance was 45%. Performances were less good mainly among CHWs from Nigeria and Uganda (Table 3). Study location and gender were co-related. Males (primarily in Burkina Faso) were 5.5 times more likely to have a good performance than females (odds ratio [OR], 5.5; 95% confidence interval [CI], 3.24–9.35; *P* < .0001), and the odds of performing well was 3 times greater for the CHWs <30 years old compared with older CHWs (Table 4). Single CHWs performed better than married CHWs (OR, 0.27; 95% CI, .09–.83; *P* = .0197), and CHWs with <1 year of experience did better than those who had 1–3 years’ experience (OR, 3.71; 95% CI, 1.66–8.29; *P* = .0012). CHW farmers/pastoralists were 3 times more likely to have good performances compared with those who were in other occupations (OR, 2.94; 95% CI, 1.06–8.08; *P* = .0368) (Table 4).

**Table 3.** Performance of Community Health Workers in Assessing, Classifying, and Prescribing Treatment and Counseling for Children With Malaria of Various Degrees of Severity^a^

Performance	Burkina Faso	Nigeria	Uganda	All Countries
Assess				
Poor performance	0 (0)	2 (5.71)	2 (1.45)	4 (1.50)
Medium performance	2 (2.13)	25 (71.43)	94 (68.12)	121 (45.32)
Good performance	92 (97.87)	8 (22.86)	42 (30.43)	142 (53.18)
Classify				
Poor performance	1 (1.06)	4 (11.4)	5 (3.62)	10 (3.75)
Medium performance	0 (0)	0 (0)	0 (0)	0 (0)
Good performance	93 (98.94)	31 (88.57)	133 (96.38)	257 (96.25)
Treat and counsel				
Poor performance	9 (9.570)	18 (51.43)	117 (84.78)	144 (53.93)
Medium performance	0 (0)	6 (17.14)	10 (7.25)	16 (5.99)
Good performance	85 (90.43)	11 (31.43)	11 (7.97)	107 (40.07)
Overall				
Poor performance	0 (0)	2 (5.71)	4 (2.90)	6 (2.25)
Medium performance	3 (3.19)	23 (65.71)	116 (84.06)	142 (53.18)
Good performance	91 (96.80)	10 (28.57)	18 (13.04)	119 (44.57)

Data are presented as No. (%). Performance scores: poor, <60%; medium, 60%–89%; and good, ≥90%.

^a^ In the assessment, most (173/220) community health workers (CHWs) were observed only once. However, 47 CHWs were observed in the management of 2 sick children.

**Table 4.** Association Between Community Health Workers' Sociodemographic Characteristics and Their Performance**^a^**

Sociodemographic Characteristics of CHWs	All Observations,	Score ≥90%,	OR (95% CI)	*P* Value
	No.	No. (%)		
Sex				
Male	110	75 (68.18)	…	…
Female	157	44 (28.03)	5.50 (3.24–9.35)	<.0001
Age group				
<30 y	54	37 (68.52)	…	…
30–45 y	151	57 (37.75)	3.59 (1.86–6.91)	.0001
>45 y	55	24 (43.64)	2.81 (1.29–6.12)	.0089
Marital status				
Married	225	101 (44.89)	…	…
Not married	16	12 (75)	0.27 (.09–.83)	.0197
Other	9	3 (33.33)	1.63 (.43–6.09)	.4939
Level of education				
Primary	109	55 (50.46)	…	…
Secondary	128	59 (46.09)	1.19 (.72–1.98)	.5027
Experience as CHW				
<1 y	39	25 (64.10)	…	…
1–3 y	77	25 (32.47)	3.71 (1.66–8.29)	.0012
>3 y	148	69 (46.62)	2.04 (.99–4.20)	.0521
Main occupation				
Farmer/animal husbandry	194	96 (49.48)	…	…
Trader	47	18 (38.30)	1.58 (.83–3)	.1681
Other	20	5 (25)	2.94 (1.06–8.08)	.0368

Abbreviations: CHW, community health worker; CI, confidence interval; OR, odds ratio; y, year.

^a^ In the assessment, most (173/220) CHWs were observed only once. However, 47 CHWs were observed in the management of 2 sick children.

## DISCUSSION

This study provides quantitative evidence that among 331 CHWs trained to assess, classify, treat, and counsel parents of sick children, 220 when assessed later performed acceptably in assessing and treating young children; there were site differences and substantial variance in performance, being consistently better in Burkina Faso than elsewhere. Young, single, less experienced CHWs performed better than older, married, more experienced CHWs.

Some challenges were faced in training. In Burkina Faso, it proved difficult to find literate CHW candidates, and some candidates proposed by their communities did not pass the training qualifications, which required minimum numeracy and literacy to complete documentation. Age calculations were difficult; because medicine dosages were based on age, incorrect calculations would risk patient safety. Close supervision and working through cases enabled progressive improvements through a continuous education process. Some CHWs suggested longer training periods to improve. This is not always possible, and training for malaria control often occurs before or during the rainy season, which can be counterproductive if CHWs are concerned about their farm work. Training CHWs too early before their deployment can cause problems if this increases the risk of losing skills acquired during training. In some countries, we chose CHWs who had their own drug shops; this can either create a conflict of interest or be advantageous if there are many ruptures in stock.

After almost 1 year of implementation, the performance of CHWs in assessing a child's condition was reasonable. All CHW adequately took the history of the child. Respiratory rates were often underestimated by CHWs as compared with observers. This might be a result of the difficulties in keeping the child calm during an examination, which highlights the need to support new CHWs during their assessments and to develop the habit of recording values immediately. RDT proficiency was good, with almost all the readings being in agreement with those of observers. Good performance has been reported in the literature and appears to be influenced by the use of the job aids during practice [20, 21].

CHWs are chosen by their communities. A positive association between good performance and young, single status shown in this study is probably a consequence of the fact that young male CHWs were mainly in Burkina Faso where there was superior overall performance. Importantly, in Burkina Faso, not all CHWs passed certification for severe malaria. Those that were not certified were not provided with rectal artesunate. In addition, in Burkina Faso, CHWs were allowed to retail ACTs at a nominal price, which may have played a role in their performance compared with the other countries. It is interesting that education was not significantly associated with good performance, suggesting that the tasks do not necessarily depend upon education level, except for literacy and numeracy.

It should be pointed out that poststudy evaluations took place at the health facility level, which could have influenced performance. In addition, the observers knew the results of CHWs before documenting their own assessments. We tried to minimize the bias this might involve by close observation. Finally, the study involved only CHWs who completed the study, all of whom had passed a certification examination before deployment and were closely supervised during the study. No misuse of drugs was found in drug accountability.

This study provides evidence that CHWs can perform well when well trained. They should be certified before being deployed, have refresher training, and be closely supervised after training to increase confidence in their performance.

## Supplementary Data

Supplementary materials are available at http://cid.oxfordjournals.org. Consisting of data provided by the author to benefit the reader, the posted materials are not copyedited and are the sole responsibility of the author, so questions or comments should be addressed to the author.

Supplementary Data
