# Supplementary material for: Training Community Health Workers to Manage Uncomplicated and Severe Malaria: Experience From 3 Rural Malaria-Endemic Areas in Sub-Saharan Africa
Source: Clin Infect Dis. 2016 Dec 6;63(Suppl 5):S264–9. doi: 10.1093/cid/ciw624 (PMC5146696; doi:10.1093/cid/ciw624)
Supplement: Supplementary Data [file supp_63_suppl-5_S264__index.html]

Supplementary Data 

# Training Community Health Workers to Manage Uncomplicated and Severe Malaria: Experience From 3 Rural Malaria-Endemic Areas in Sub-Saharan Africa

## Supplementary Data

Supplementary Data

- Supplementary Data - Pdf file
