# Supplementary material for: Training Community Health Workers to Manage Uncomplicated and Severe Malaria: Experience From 3 Rural Malaria-Endemic Areas in Sub-Saharan Africa
Source: Clin Infect Dis. 2016 Dec 6;63(Suppl 5):S264–9. doi: 10.1093/cid/ciw624 (PMC5146696; doi:10.1093/cid/ciw624)
Supplement: Supplementary Data [file supp_ciw624_ciw624supp.pdf]

Supplementary Table 1: Training models and topics covered in Burkina Faso

Who was trained and job description	Trainers	Duration of the training	Content of training	Supervisors
Nurses<br>Train and supervise community health workers	Investigative team* and district health team	Two days	· Signs and symptoms of uncomplicated and severe malaria<br>· Identification of eligible patients for uncomplicated malaria treatment and differentiation of patients to be treated with oral drugs vs those to be treated with rectal artesunate<br>· Obtaining informed consent from parent/ guardian<br>· Use of RDTs and interpretation of results<br>· Counting respiratory rates<br>· Measuring temperature<br>· Administration of treatment<br>· Referral of patients with danger signs to health centres<br>· Follow – up of each RA treated patient<br>· Record keeping and bookkeeping<br>· Adverse events reporting<br>· Monitoring and supervision<br>· Conduct of educational talks	–
Drugs store manager<br>Drugs storage, drugs distribution to community health workers, records keeping.	Nurses	Three days	· Signs and symptoms of uncomplicated malaria and severe malaria<br>· Identification of danger signs<br>· Obtaining informed consent from parent/ guardian<br>· Use of RDTs and interpretation of results<br>· Counting respiratory rates<br>· Measuring temperature<br>· Administration of treatment<br>· Referral of patients with danger signs to health centres<br>· Follow – up of each RA treated patient<br>· Record keeping and book keeping<br>· Adverse events reporting<br>· Use of Pictorials for CCC	Investigative team and district health team
Community health workers	Nurses	Three days	· Signs and symptoms of uncomplicated malaria and severe malaria<br>·Identification of danger signs<br>· Obtaining informed consent from parent/ guardian<br>· Use of RDTs and interpretation of results<br>· Counting respiratory rates<br>· Measuring temperature<br>· Administration of treatment<br>· Referral of patients with danger signs to health centres<br>· Follow – up of each RA treated patient<br>· Record keeping and book keeping<br>· Adverse events reporting<br>· Counselling of caregivers<br>· Use of Pictorials for IEC<br>· Conduct of educational talks	Investigative team and district health team

*The investigative team was multidisciplinary composed of physicians, medical biologist, socio – anthropologists and epidemiologist

Supplementary Table 2: Training models and topics covered in Nigeria

Who was trained and job description	Trainers	Duration of the training	Content of training
Training of Community health workers	Investigative team* and district health team	3 days	Causes of fever<br>Malaria (causes & transmission)<br>Signs & symptoms of UC/severe malaria<br>Practical session on use of RDTs<br>Presumptive diagnosis vs. RDT<br>Preparation of thick blood smears<br>Record keeping<br>Completion of registers<br>Other data collection tools<br>Case management & referral<br>Communication skills/ethics<br>Accountability for drugs & RDTs

* specialists in epidemiology, clinical pharmacology, clinical, medical sociology, anthropology, health education, laboratory techniques and environment health

Supplementary Table 3: Training models and topics covered in Uganda

Who was trained and job description	Trainers	Duration of the training	Content of training
ICCM workshop of Community health workers	Investigative team** and district health team	Five days	Role of the CHW<br>Clinical practice: Asking the caregiver about the child's problems<br>Identify fast breathing<br>Identify danger signs<br>Referring a sick child or new born to a health facility<br>Clinical practice for assessment, classification, referral<br>RDT use and interpretaion<br>Introduction to the study on follow up of children<br>Treat child with no danger signs<br>Treat fever, cough, diarrhoea<br>Pre-referral treatment<br>Routine newborn care<br>Medicine management

* total 234 CHWs passed the post training test but not all of them became really active study CHWs

** The investigative team was composed of physicians, socio – anthropologists and epidemiologists

Supplementary Table 4: List of variables and indicators assessed

Objectives	Variables	Indicators/Training items covered - Burkina
Assess	General practice	Asking about hot body, the child's age/date of birth, calculation of child's age, temperature measurement, respiratory rate measurement, looking for danger signs
	Proficiency in the use of RDT	Safety measures, labelling cassettes, pricking the correct finger, accuracy of blood volume collected, correct volume of buffer applied, blood and buffer placed in appropriate wells, wait right time before reading the test result, interpretation of the results
Classify	Adherence to treatment recommendations and medicines dosage	RDT positive + ACT, RDT positive + no ACT, RDT negative + ACT, RDT negative + no ACT, when RA should be given
	Medication dosages	Dosage of medecines to be given
Treat and Counsel_		Medicine dosage explanations. Return within 48 hours if patient does not improve. Feed the child well.

Supplementary Table 5. **Training tools used in the study**

Training Documents	Description
Training Manuals	Burkina Faso: 1. Training manual on the management of uncomplicated malaria designed by the investigative team based on the guideline of the National Malaria Control Programme of Burkina and the Manual of WHO for the training of CHWs.<br>2. Simplified version of the WHO Manual for pre - referral treatment with Rectal Artesunate – French version (http://video.who.int/streaming/tdr/WHO-TRA_Malaria_rectal_artesunate_19SEPT2012_fr.wmv)<br>Nigeria: 1. Simplified versions of WHO manuals for the training of CHW on uncomplicated malaria. 2. WHO Manual for pre - referral treatment with Rectal Artesunate – English version. (http://video.who.int/streaming/tdr/WHO-TRA_Malaria_rectal_artesunate_19SEPT2012_en.wmv)<br>Uganda: 1. Simplified versions of WHO manuals for the training of CHW on uncomplicated malaria. 2. WHO Manual for pre - referral treatment with Rectal Artesunate – English Version.
Job Aids*	On the use of Rapid diagnostic test of malaria (mRDT) developed in French in Burkina Faso, Yoruba in Nigeria and Luganda and Lunyankole in Uganda, based on the job aids of FIND.
Informed Consent Form	The three study sites (Burkina Faso, Nigeria, Uganda) used the Ethical committee approved Participant Information Sheets and the Informed Consent Forms developed for the purpose of the study.
Case Report Forms	The three study sites used the Case Report Forms (CRFs) developed for the purpose of the study:<br>Patient register used for uncomplicated malaria and as screening form for severe malaria<br>Pictorial Recruitment form for severe malaria (Rectal Artesunate group)<br>Two follow-up forms for patients recruited in rectal artesunate group
Drugs Accountability Forms	The three study sites used the rectal artesunate drug accountability forms designed for the purpose of the study (one for the central level, one form at health facility level and one form at peripheral level (CHWs). Each form was checked against CRFs to monitor drug accountability at each supervisory visit
Referral Card	To accompany a patient being referral to the nearest health facility for patients who received rectal artesunate.
Treatment Information Material*	Burkina Faso: Two posters on Artemether - Lumefantrine dosage according to the age groups (6-36months and 36 - 59 months), one poster on the advantages of correct treatment/dosage and one on the disadvantages of incorrect treatment/dosage.<br>Pictorials for danger signs and for the methods of Rectal Artesunate Administration<br>Nigeria: A mannequin for explaining and practice on the insertion of Rectal Artesunate.<br>Uganda: Integrated Management of Childhood Illness materials developed

*The printable materials, job aids were laminated for each CHWs for their training and for communication, sensitization of caregivers during the consultation, the community during educational talks for behavior changes.
